# Remodeling the Dendritic Spines in the Hindlimb Representation of the Sensory Cortex after Spinal Cord Hemisection in Mice

**DOI:** 10.1371/journal.pone.0132077

**Published:** 2015-07-01

**Authors:** Kexue Zhang, Jinhui Zhang, Yanmei Zhou, Chao Chen, Wei Li, Lei Ma, Licheng Zhang, Jingxin Zhao, Wenbiao Gan, Lihai Zhang, Peifu Tang

**Affiliations:** 1 Department of Orthopaedics, General Hospital of Chinese PLA, Beijing, 100853, People’s Republic of China; 2 Drug Discovery Center, Key Laboratory of Chemical Genomics, Peking University Shenzhen Graduate School, Shenzhen, 518055, People’s Republic of China; 3 Department of Physiology and Neuroscience, New York University School of Medicine, New York, New York, 10016, United States of America; University of South Alabama, UNITED STATES

## Abstract

Spinal cord injury (SCI) can induce remodeling of multiple levels of the cerebral cortex system especially in the sensory cortex. The aim of this study was to assess, *in vivo* and bilaterally, the remodeling of dendritic spines in the hindlimb representation of the sensory cortex after spinal cord hemisection. Thy1-YFP transgenic mice were randomly divided into the control group and the SCI group, and the spinal vertebral plates (T11–T12) of all mice were excised. Next, the left hemisphere of the spinal cord (T12) was hemisected in the SCI group. The hindlimb representations of the sensory cortex in both groups were imaged bilaterally on the day before (0d), and three days (3d), two weeks (2w), and one month (1m) after the SCI. The rates of stable, newly formed, and eliminated spines were calculated by comparing images of individual dendritic spine in the same areas at different time points. In comparison to the control group, the rate of newly formed spines in the contralateral sensory cortex of the SCI group increased at three days and two weeks after injury. The rates of eliminated spines in the bilateral sensory cortices increased and the rate of stable spines in the bilateral cortices declined at two weeks and one month. From three days to two weeks, the stable rates of bilaterally stable spines in the SCI group decreased. In comparison to the control group and contralateral cortex in the SCI group, the re-emerging rate of eliminated spines in ipsilateral cortex of the SCI group decreased significantly. The stable rates of newly formed spines in bilateral cortices of the SCI group decreased from two weeks to one month. We found that the remodeling in the hindlimb representation of the sensory cortex after spinal cord hemisection occurred bilaterally. This remodeling included eliminating spines and forming new spines, as well as changing the reorganized regions of the brain cortex after the SCI over time. Soon after the SCI, the cortex was remodeled by increasing spine formation in the contralateral cortex. Then it was remodeled prominently by eliminating spines of bilateral cortices. Spinal cord hemisection also caused traditional stable spines to become unstable and led the eliminated spines even more hard to recur especially in the ipsilateral cortex of the SCI group. In addition, it also made the new formed spines unstable.

## Introduction

Spinal cord injury (SCI) dramatically alters sensory and motor functions by seriously blocking the normal function of sensory inputs and motor outputs between the brain and the body [1–3]. Significant changes occur in the way in which the relevant sensory cortex receives sensory inputs from the body after SCI [4]. In addition to the direct loss of motor and sensory functions, SCI can induce massive long-term remodeling of the brain up to the relevant motor and sensory cortices [1,5–7]. Long-term cortical reorganization may lead to a certain amount of recovery on selective motor and sensory functions. However, maladaptive or abnormal reorganization can induce illusory sensations that do not reflect objective reality, such as neuropathic pain [8–11], hyperpathia, and phantom sensations [5].

Assessing reorganization and exploring the underlying mechanisms of cortical remodeling after SCI are important goals for administering efficient and timely interventions to properly modulate the pathological and physiological consequences and to obtain the best recovery outcomes [12]. Several mechanisms may contribute to cortical reorganization after SCI. These include alterations in the intrinsic neural properties [6], the appearance of new connections due to dendritic and axonal sprouting [13,14], and the appearance of underlying neural connections due to a decrease in intracortical inhibition [15]. The data from two recent, *in vivo* studies have demonstrated that different sensory experience significantly affects the stability of spines in various cortices by observing over time the dynamics of yellow and green fluorescent protein (YFP and GFP) labeled dendritic spines [16,17]. The reorganization of the cortex after SCI may be driven by the same mechanisms as learning and memory and also may lead to remodeling of the cortical dendrite spines. Dendritic spines are the postsynaptic component of the majority of excitatory synapses in the central nervous system [18,19], and are significant indicators about the remodeling of synaptic connectivity [20–22]. They contain all of the essential components for postsynaptic signal transmission and plasticity. Both the population and morphology of dendritic spines are sensitive to different types of sensory experience and environmental stimuli [23,24]. Several studies have reported the reorganization of the motor cortex in pathological conditions such as limb amputations and peripheral nerve injuries [25–27]. Kim *et al*. discovered that spine density decreased at seven days after SCI and partially recovered by 28 days after SCI in the motor cortex [28]. Ghosh *et al*. found that spine loss occurred on the apical dendrite of axotomized neurons and non-axotomized layer 5b neurons in the denervated hindlimb cortex after SCI [29].

However, these studies were performed with functional magnetic resonance imaging, electrophysiology, or confocal microscopy with fixed cortical slices. Functional magnetic resonance imaging and electrophysiology are only able to assess the remodeling of the brain after SCI at a low resolution. Furthermore, the methods that require slicing are limited to assessing the net of spine elimination and formation, but they cannot distinguish what happen between those processes over time after SCI. These methods can show portions of the changes in spine density and morphology, but cannot be used to repeatedly image individual dendritic branches and spines in the same animal. Thus, previous researches could not determine definitively how dendritic spines are remodeled after SCI. Three major questions remain to be resolved: (1) Are there differences between the bilateral hindlimb sensory cortices after spinal cord hemisection and what are they? (2) Does cortex remodeling occur through spine elimination and spine formation at distinctive time points? (3) What happen among traditional stable spines, eliminated spines and newly formed spines after SCI? Are they still stable or eliminated and does the eliminated spines recur in the long run?

Advances in high-resolution, time-lapse imaging, together with transgenic fluorescent molecular tools, have made it possible to repeatedly and longitudinally image individual dendritic branches and spines in the same animal at different times. Performing two-photon microscopy on transgenic mice [30] in which a small population of neurons are YFP-labeled permits the observation and measurement of the turnover rates and morphological changes of the dendritic spines in many cortical regions during growth, experience, learning, and memory [31–34]. In this study, transcranial two-photon microscopy (thinning skull protocol) and Thy1-YFP transgenic mice (YFP mice) that expressed predominantly in the layer V pyramidal neurons were used to image repeatedly individual dendritic branches and spines in the hindlimb sensory cortex after the SCI. The hindlimb cortices were observed bilaterally after spinal cord hemisection to compare the changes between them. The aims of this experiment were (1) to determine whether spinal cord hemisection leads to bilateral remodeling of the synaptic structures in the sensory cortex, (2) to assess the differences between the bilateral sensory cortices of hindlimb after spinal cord hemisection, (3) to determine whether the remodeling occurs through spine elimination or spine formation at distinctive time points, (4) to determine what happen among the traditional stable spines, eliminated spines and newly formed spines after the SCI and to discover whether them are stilly stable, or be eliminated or recur in the long run.

## Materials and Methods

### Animals

Clean, healthy, male, Thy1-YFP transgenic mice that express YFP in the layer V pyramidal neurons (H–line, certification No. 2013–0002), 30 days old, and weighing 11–14 g were purchased from Jackson Laboratory (Bar Harbor, ME, USA) and bred in the Experimental Animal Center in the Shenzhen Graduate School of Peking University. The mice were housed in standard cages at a constant temperature of 21–25°C and humidity of 50–60%. They were fed a normal diet and were given free access to water. This study was carried out in strict accordance with the recommendations in the Guide for the Care and Use of Laboratory Animals of the National Institutes of Health. The protocol was approved by the Committee on the Ethics of Animal Experiments of Chinese PLA General Hospital. All surgeries were performed under the anesthesia of sodium pentobarbital and all efforts were made to minimize suffering. ‘N’ in the article means the number of mice. Sodium pentobarbital (10.0 ml/kg body weight) at 100 mg/kg in 0.9% NaCl was used to anesthetize mice during all surgical procedures. The condition of the animals was monitored each day. We checked each day general appearance, posture, coat condition, responses to handling, demeanor, diet information and body weight of each mouse. The mice were also carefully scrutinized to make sure that there were no infections or injuries. During the experiment, one mouse in the SCI group was found dead unexpectedly on 15th day after the SCI. This might have been due to inappetence and decreasing body weight (a weight loss 15.4% on the 15th day after the SCI). There were three mice that were euthanatized by excess injection of sodium pentobarbital during the experiment for accidentally damaging the cortices at the third or fourth time of the thinning skull. At the end of the experiment, all mice were euthanatized by excess injection of sodium pentobarbital.

### Spinal cord hemisection

**Fig 1:**
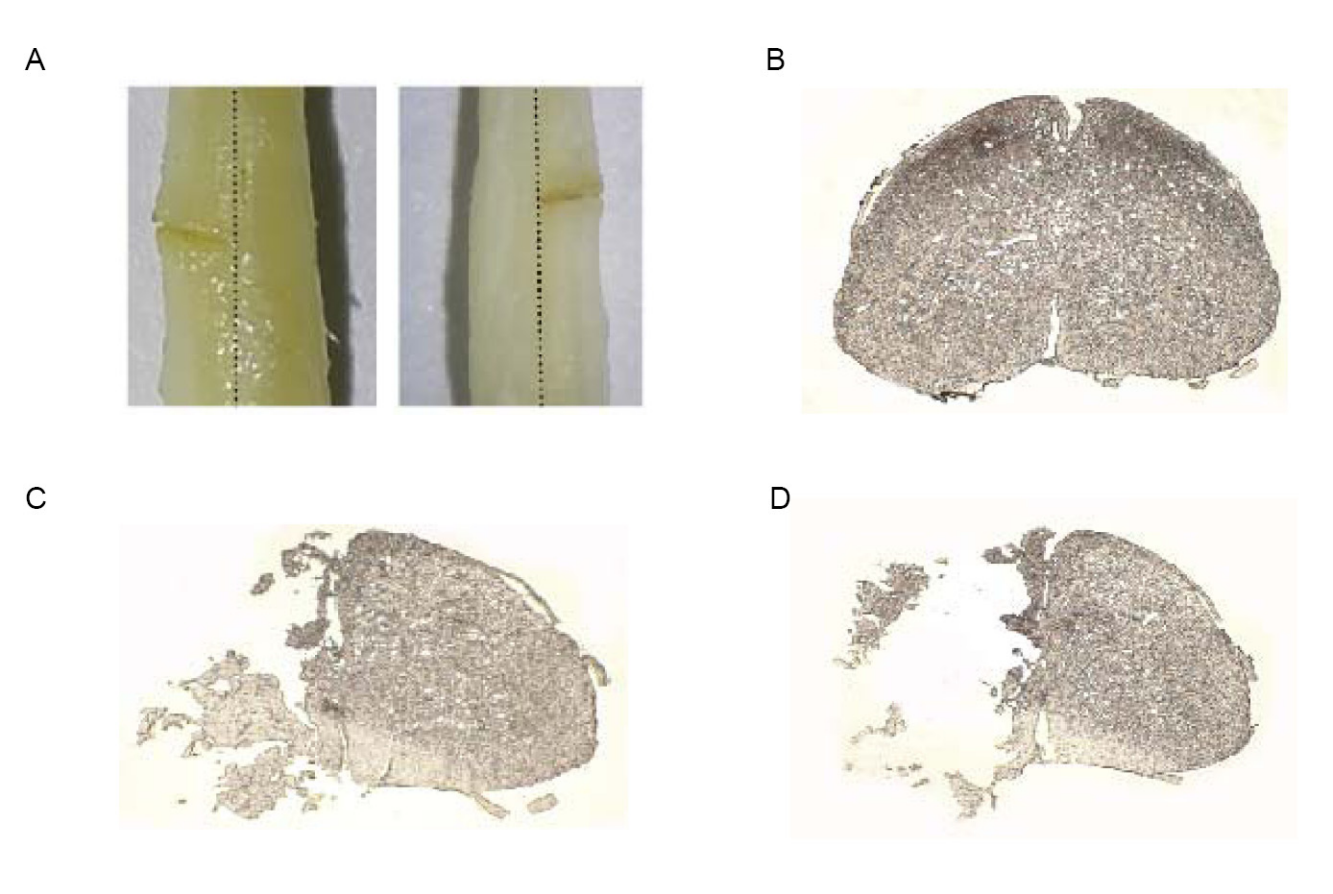
Spinal cord hemisection (T12). (A) Representative hemisection in left hemisphere of the spinal cord (dorsal and ventral views). (B) Intact section of spinal cord. (C and D) Schematic representative section of hemisection in the left hemisphere of the spinal cord.

The thoracic spinal cord hemisection procedures in mice were similar to those described in previous studies [14,35,36]. The spinal cord hemisection was achieved by hemisecting the left hemisphere of the spinal cord (T12) in the YFP mice under anesthesia by an intraperitoneal injection of sodium pentobarbital (10.0 ml/kg body weight) at 100 mg/kg in 0.9% NaCl. Briefly, after laminectomy of the spinous process and lamina of the T11–T12 vertebrae, the spinal cord was hemisected on the left hemisphere from the dorsal in the ventral direction at the T12 level with a microblade. All operations were performed under an anatomical microscope. To ensure complete hemisection, the spinal cord was cut twice. The transection was also visually confirmed by the entire separation of the transected terminals. At the same time, three mice were euthanized under deep anesthesia after the SCI by an intraperitoneal injection of sodium pentobarbital (10.0 ml/kg body weight) at 100 mg/kg in 0.9% NaCl and perfused with paraformaldehyde (4%) to fix the spinal cords for histological analyses (Fig 1). Sham operations were also performed by performing laminectomy of the T11–T12 spinal vertebral segments without the left hemisection of the spinal cord. Following this, the incised muscle and skin were sutured and the mice received postoperative care, they were placed onto the heating blanket (37°C) where they remained until they had become totally conscious. All mice were subcutaneously injected with 0.2 mL 0.9% NaCl in order to avoid dehydration. In addition, an antibiotic was administered intraperitoneally to prevent infection during the experiment. The urinary bladders of mice were emptied manually twice per day until automatic micturition function recovered. To relieve pain following surgery, paracetamol (200 mg) was dissolved in drinking water (100 mL) in the first day after injury. It gave an average drug dosage of about 100 mg/kg b.w./day.

### Histological Analysis

Three mice were sacrificed following spinal cord hemisection and used to indicate the histological examination of the lesion size after the SCI. The mice were deeply anesthetized and perfused with paraformaldehyde(4%) after spinal cord hemisection. The spinal cords were then removed and post-fixed in paraformaldehyde(4%). Next, they were immersed in sucrose (10%) overnight and then transferred into sucrose(30%) for 24 hours. The spinal cords were then embedded into Optimal Cutting Temperature (OCT) compound and sectioned with a cryostat to obtain axial sections of a thickness of 10 μm. The images of sections were examined and obtained by transmitted light microscopy (Fig 1B–1D).

### *In vivo* transcranial two-photon imaging

#### Mouse head grinding and fixation

**Fig 2:**
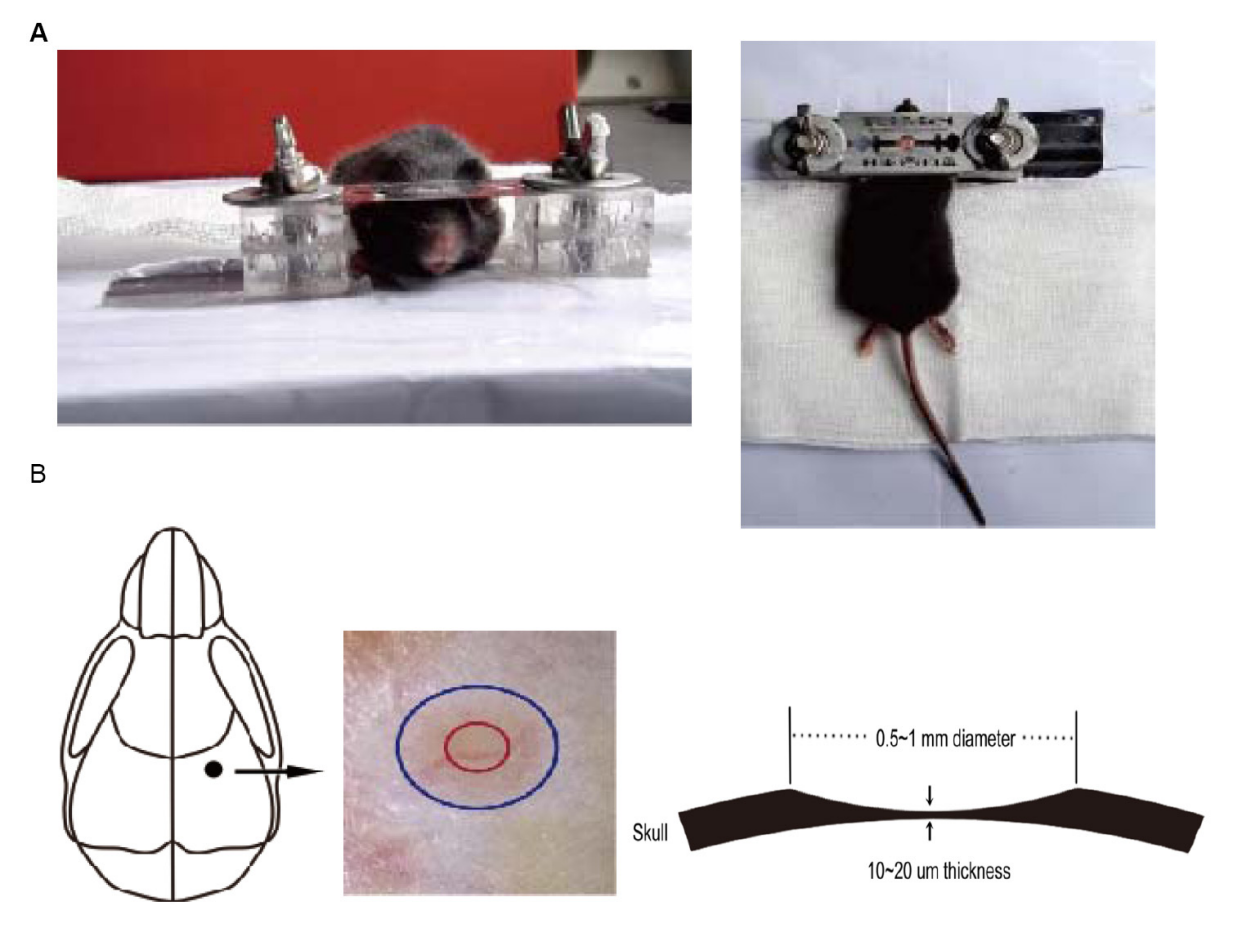
Schematic of thinned skull preparation. (A)The immobilization device for the mouse’s head, which includes a skull holder and a custom-built plate, is used to minimize the movement artifacts when imaging (view from the front and above). (B) The targeted, thinned area of the skull is marked with red circle for in vivo TPLSM imaging. The thinnest area is about 20 μm in thickness and 200 μm in diameter.

Under anesthesia with an intraperitoneal injection of sodium pentobarbital (10.0 ml/kg body weight) at 100 mg/kg in 0.9% NaCl, the scalp of each mouse was incised along the middle of the skull to expose the desired area. Representative regions of the hindlimb primary sensory cortices were stereoscopically localized, following the previous methods [37]. A stainless steel sheet was fixed onto the observed area with a cyanoacrylate adhesive to minimize respiration-induced movements. The steel sheet had a gap in the middle that allowed easy observation of the desired area. Next, the mouse and steel sheet were secured to two lateral bars that were located on both sides of the mouse head and fixed to a metal base (Fig 2A). Under observation with a microscope, a high-speed drill was used to grind the observed region of the mouse head. The thickness of the skull was reduced carefully by more than 50%. During the grinding, an artificial cerebrospinal fluid was intermittently dropped onto the observed region to prevent frictional heat-induced intracranial tissue damage. The thinning operation was completed by scraping the surface of skull with a micro-blade (Surgistar no. 6400). For best quality of imaging, the thickness of skull was reduced to about 20 μm. Two-photon microscopy detection was used to ensure that a thickness of ~20μm was achieved. The grinding procedure lasted approximately 30 minutes. After grinding, the mouse was imaged. Then the steel sheet was carefully removed. After being cleaned and disinfected, the mouse was sutured with 6–0 suture, placed onto a heating blanket until it had regained consciousness, and then housed in a cage until the next imaging day. All mice were subcutaneously injected with 0.2 mL 0.9% NaCl in order to avoid dehydration. An antibiotic was administered intraperitoneally to prevent infection during the experiment.

### Identification of the hindlimb region of the sensory cortex

**Fig 3:**
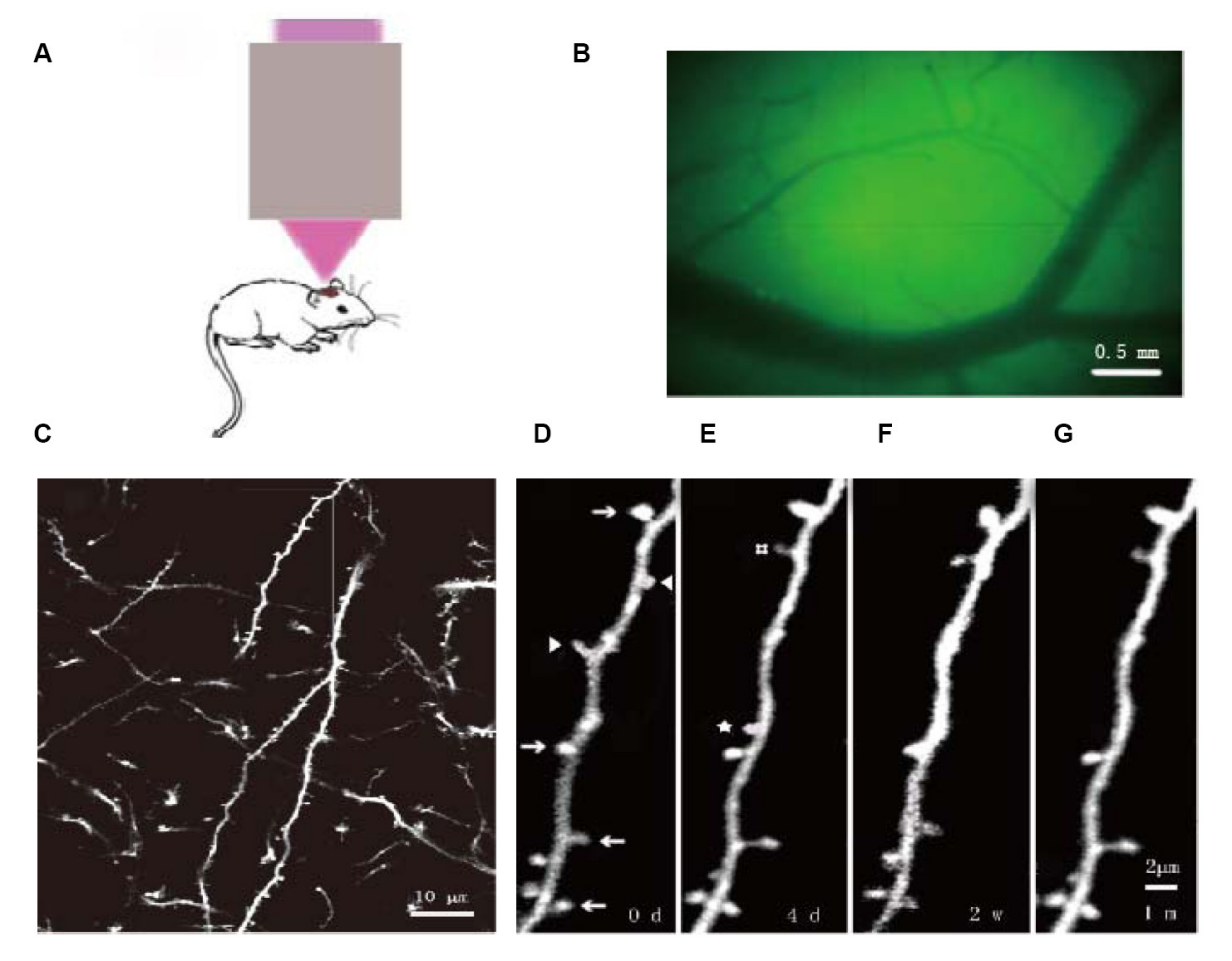
Dendritic spines of YFP mice longitudinally observed by two-photon microscopy. (A) Schematic of the imaging of the cortex using the two-photon carrier. (B) Locating the observation site by viewing vasculature under the thinned skull. (C) In vivo imaging the primary sensory cortex at 25× magnification, the dendritic spines and axons appear clearly. (D–G) Magnified images of (C) at (D) day 0 and (E) day 4, at (F) two weeks, and at (G) one month. Arrows: dendritic spines observed at the indicated four time points. Arrowheads: dendritic spines that disappeared on the fourth day. #: dendritic spines that were newly formed by the fourth day and remained at two weeks and one month. ☆: dendritic spines that were newly formed by the fourth day, but disappeared at two weeks. Scale bars: 0.5mm(B),10 μm (C), 2 μm (D, E, F, G).

The hindlimb representation of the sensory cortex region was located 1.4 mm posterior to the bregma and within 1.7 mm laterally from the midline. Those regions have been identified by microstimulation as the location of the hindlimb sensory representative region in the same mouse strain as that used in the present experiments. The hindlimb representation region was confirmed by cytochrome oxidase staining. The imaging window used here was small (~200 μm in diameter, Fig 2B).We used stereotaxic coordinates of previously mapped hindlimb sensory regions for guidance into studying the dynamics of the dendritic spine in the sensory cortex [33,38–40].The vasculature pattern that was indicated by anatomical microscope (Fig 2B) and two-photo microscope (Fig 3B) remained stable for months to years and could be used as a landmark to relocate the image area in the sensory cortex at three subsequent time points.

### Two-photon imaging of dendritic spines *in vivo*

The imaging methods used here were similar to those used in previous studies [32,37,41,42]. During imaging, after the mouse was anaesthetized by an intraperitoneal injection of sodium pentobarbital (10 ml/kg body weight) at 100 mg/kg in 0.9% NaCl, the head of the mouse with the steel attached was secured to a special fixture and the observation region was placed under the two-photon microscope to reduce respiratory interference with imaging (Fig 2A). The imaging conditions were as follows: (1) excitation wavelength: 920 nm; (2) scan mode: XYZ; (3) objective lens: 25× hydroscope; (4) laser power: < 40 mW; (5) scan intensity: 10 to 20%; (6) scan depth < 150 μm; (7) scan thickness: 2 μm and 0.75 μm; and (8) ZOOM values: 1 and 3. To facilitate multiple resolution imaging, the scan thickness was first set at 2 μm at a low magnification (zoom = 1) for the first scan to determine the overall location of the desired observation area. Then, the scan thickness was decreased to 0.75 μm (zoom = 3) for image acquisition.

### Image quantification

Data analysis was performed as described previously [32,34,41,43,44]. A total of 150–200 randomly selective dendritic spines were observed in each mouse. Slender, non-enlarged head dendritic protrusions (twice as long as normal dendritic spines, head radius/neck radius of protrusion < 1.2, and total length/neck radius of protrusion > 3) were classified as filopodia [43]. The remaining dendritic protrusions were classified as dendritic spines. Images were collected at the same position and of the same dendrite spines at each time point. Dendritic spines were considered stable between two images on the basis of their spatial relationship to adjacent landmarks and their position to adjacent dendritic spines. Dendritic spines in the second image were considered to be different (newly formed or eliminated) if they were > 0.7 μm from their expected positions based on the first image (Fig 3D–3G). The numbers of newly formed, eliminated, and unchanged or stable dendritic spines were calculated by comparing images of the same position at different time points and using the following formulas:

1. The rates of newly formed or eliminated dendritic spines = the number of formed or eliminated dendritic spines / the number of dendritic spines in the first image.
2. The stable rate = the number of stable dendritic spines exist in two images / the number of dendritic spines in the first image.
3. The stable rate of stable spines (e.g. three days to two weeks) = the number of stable dendritic spines that exist in images at the three times (0d, 4d and 2w) / the number of stable spines exist in the first two images (0d and 4d).
4. The stable rate of formed spines(e.g. three days to two weeks) = the number of formed spines that there are in three times image (0d, 4d and 2w) / the number of formed spines that there are in the first two images (0d and 4d).
5. The re-emerging rate of eliminated spines (e.g. three days to two weeks) = the number of eliminated spines between the first two images (0d and 4d) that recur in the third time image (2w) / the number of eliminated spines in the first two images (0d and 4d, Fig 3).

### Quantitative analysis of experimental animals

**Fig 4:**
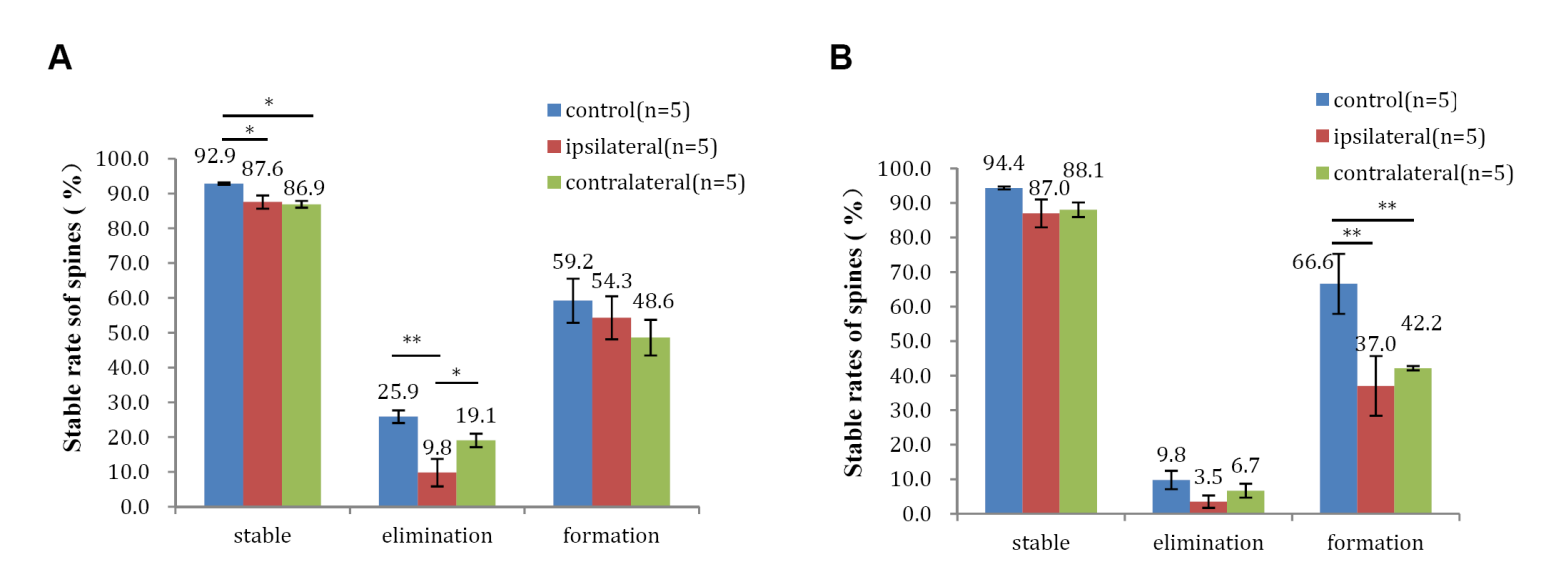
Differences in stable rates of dendritic spines between the bilateral sensory cortices of the hindlimb after spinal cord hemisection. Stable rates of (A) 3d-2w, (B) 2w-1m. All data are presented as the mean ± SEM. n: the number of mice. *: *P* < 0.05, **P < 0.01 vs each other.

To investigate the effect of spinal cord hemisection on dendritic spines in the hindlimb sensory cortex over time, we repeatedly imaged dendritic protrusions in the bilateral hindlimb representations of the sensory cortex on the day before, three days, two weeks, and one month after the SCI. The rates of stable, newly formed, and eliminated spines were calculated by comparing images of the same areas at different time points. The results between bilateral cortices in the control group showed no significant difference (Fig 4). So the results of the control group are the averages of the data from the bilateral cortices. One or two mice in each group were excluded from analysis, because of damage to the cortex during the third or fourth thinning of the skull or because they died unexpectedly during the experiment.

### Statistics

All data were analyzed with SPSS 17.0 software (SPSS, Chicago, IL, USA) and are presented as the mean ± SEM. The student's *t*-tests (symmetric distribution) or the Mann-Whitney test (asymmetric distribution) was used to compare the two groups in relation to the quantitative outcomes. When evaluating the three groups, one-way ANOVA analysis (symmetric distribution) or the Kruskal-Wallis test (asymmetric distribution) was applied. P-values that were less than 0.05 were considered to be statistically significant. More detailed statistic methods were presented in the results.

## Results

### Significant decrease in stable spines of bilateral cortices at two weeks and one month after the SCI

**Fig 5:**
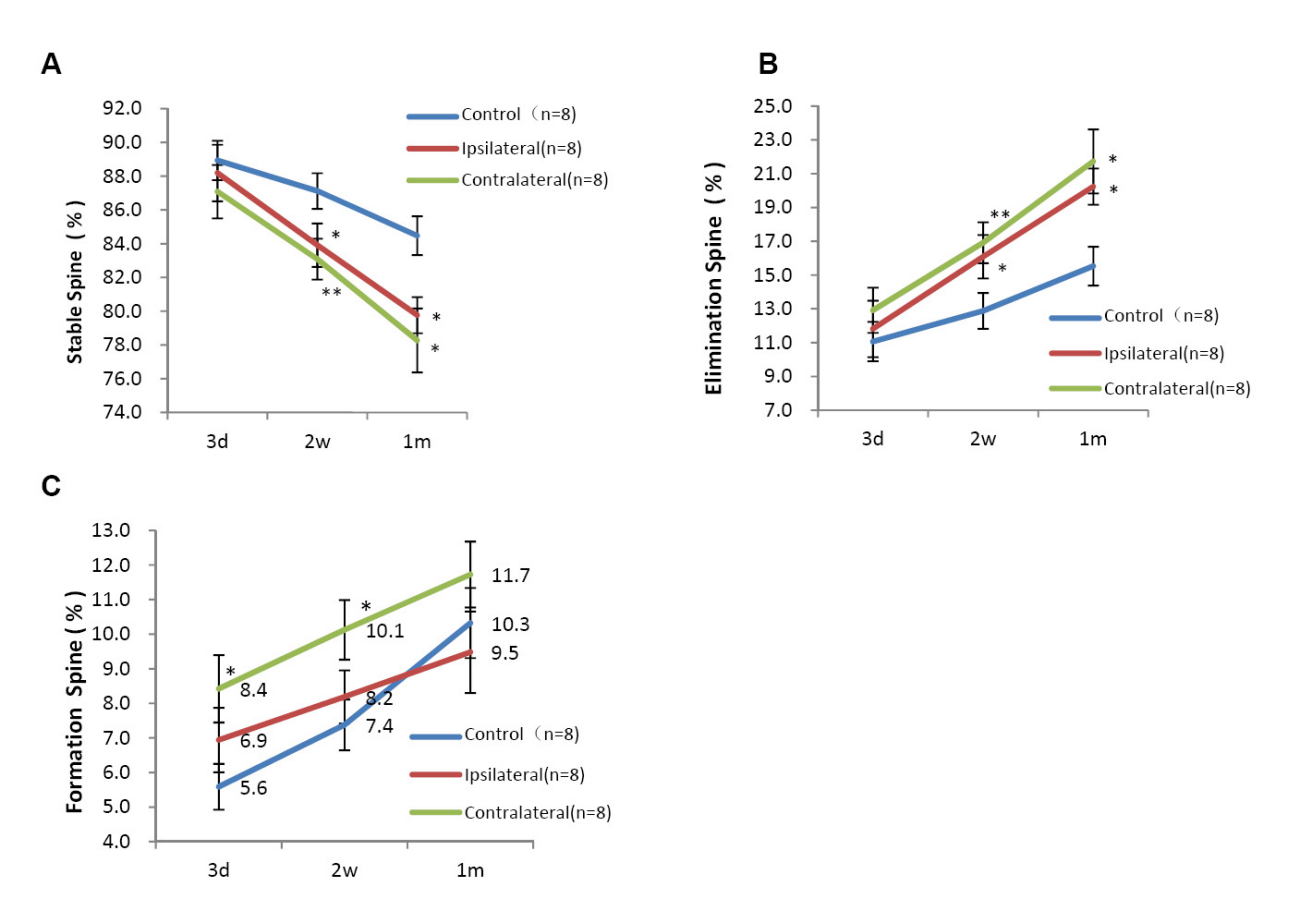
Differences in remodeling between bilateral sensory cortices of the hindlimb after spinal cord hemisection. Changes in the rates of (A) stable, (B) eliminated, and (C) newly formed spines in different groups over time. All data are presented as the mean ± SEM. n: the number of mice. *: *P* < 0.05, **P < 0.01 vs each other.

The rates of stable spines in all groups decreased over time. The rate of stable spines in the bilateral cortices at two weeks and one month were significantly lower than that of the control group (two weeks: 83.9 ± 1.3% in the ipsilateral cortex versus 87.1 ± 1.1% in the control group, *Kruskal-Wallis*, *P* < 0.05, and 83.1 ± 1.2% in the contralateral cortex versus 87.1 ± 1.1% in the control group, Kruskal-Wallis, *P* < 0.01; one month: 79.8 ± 1.1% in the ipsilateral cortex and 78.3 ± 1.9% in the contralateral cortex versus 84.5 ± 1.2% in the control group, *one-way ANOVA*, *P* < 0.05; 8 animals in each group. Fig 5A, Raw data were presented in S1–S3 Tables).

### Significant increase in spine elimination of bilateral cortices at two weeks and one month after the SCI

The rates of eliminated spines in all groups increased over time. The rates of eliminated spines in the bilateral cortices of the SCI group at two weeks and one month after injury were significantly increased compared with the control group (two weeks: 16.9 ± 1.2% in contralateral cortex versus 12.9 ± 1.1% in control group, Kruskal-Wallis, *P* < 0.01 and 16.1 ± 1.3% in ipsilateral cortex versus 12.9 ± 1.1% in control group, Kruskal-Wallis, *P* < 0.05; one month: 21.7 ± 1.9% in contralateral cortex and 20.2 ± 1.1% in ipsilateral cortex versus 15.5 ± 1.2% in control group, *one-way ANOVA*, *P* < 0.05, eight animals in each group in Fig 5B, Raw data were presented in S1–S3 Tables).

### Significant increase in spine formation of contralateral cortex at three days and two weeks after the SCI

The rates of newly formed spines in the three groups increased over time. The rates of newly formed spines in the contralateral cortex of the SCI group at three days and two weeks were significantly increased compared with those of the control group (three days: 8.4 ± 1.0% versus 5.6 ± 0.7%, Kruskal-Wallis, *P* < 0.05; two weeks: 10.1 ± 0.9% versus 7.4 ± 0.7%, one-way ANOVA, *P* < 0.05; 8 animals in each group. Fig 5C, Raw data were presented in S1–S3 Tables).

### No significant difference between the bilateral sensory cortices of the hindlimb in the control group over time

**Fig 6:**
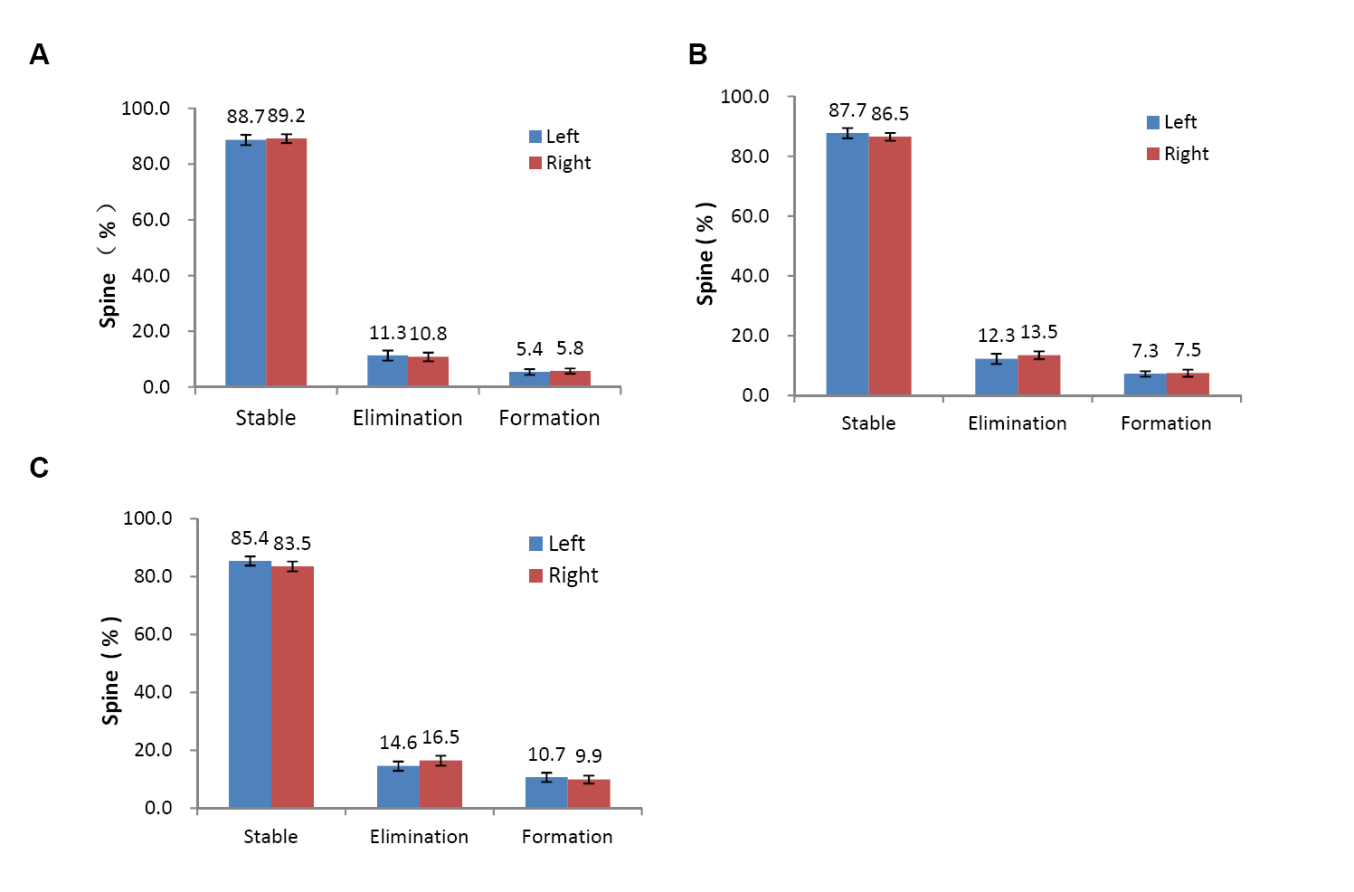
No significant difference between the bilateral sensory cortices of the hindlimb in control group over time. Remodeling of the dendritic spines in the hindlimb sensory cortex in (A) 3 days, (B) 2 weeks, and (C) 1 month. All data are presented as the mean ± SEM. n: the number of mice. * *P* < 0.05 vs each other.

The rates of stable, eliminated and newly formed spines in bilateral cortices in control group have no significant difference over one month (three days: stable spine, 88.7 ± 1.8% versus 89.2 ± 1.6%, *t-test*, *P* = 0.84,; elimination spines, 11.3± 1.8% versus 10.8 ± 1.6%, *Mann-Whitney test*, *P* > 0.05; formation spines, 5.4 ± 1.0% versus 5.8 ± 0.9%, *Mann-Whitney test*, *P* > 0.05, Fig 6A; two weeks: stable spine, 87.7 ± 1.7% versus 86.5 ± 1.3%, *Mann-Whitney test P* > 0.05; elimination spines, 12.3± 1.7% versus 13.5 ± 1.3%, *Mann-Whitney test P* > 0.05; formation spines, 7.3 ± 0.9% versus 7.5 ± 1.2%, *t-test*, *P* = 0.6569, Fig 6B; one month: stable spine, 85.4 ± 1.6% versus 83.5 ± 1.7%, *t-test*, *P* = 0.4368; elimination spines, 14.6 ± 1.6% versus 16.5 ± 1.7%, *t-test*, *P* = 0.7061; formation spines, 10.7 ± 1.6% versus 9.9 ± 1.4%, t-test, *P* = 0.725, Fig 6C).

### Significant differences in stable rates of stable and eliminated spines in the bilateral sensory cortices of the hindlimb from three days to two weeks after spinal cord hemisection

The stable rates of stable spines in bilateral cortices in the SCI group were significant lower than those in the control group (87.6 ± 1.9% in the ipsilateral cortex and 86.9 ± 1.0% in the contralateral cortex versus 92.9 ± 0.3% in the control group, *one-way ANOVA*,*P* < 0.05,n = 5, Fig 4A). The re-emerging rates of eliminated spines in ipsilateral cortices in the SCI group were significant lower than those in the control group and the contralateral cortex in the SCI group (9.8 ± 3.9% in the ipsilateral cortex versus 25.9 ± 1.8% in the control group and 19.1 ± 1.9% in the contralateral cortex, *one-way ANOVA*,*P* < 0.05, n = 5, Fig 4A).

### Significant differences in stable rates of formed spines in the bilateral sensory cortices of the hindlimb from two weeks to one month after spinal cord hemisection

The stable rates of formed spines in bilateral cortices in the SCI group were significantly lower than those in the control group (37.0 ± 8.6% in the ipsilateral cortex and 42.2 ± 0.6% in the contralateral cortex versus 66.6 ± 8.7% in the control group, *Kruskal-Wallis*,*P* < 0.01,n = 5, Fig 4B).

## Discussion

Structural remodeling at the level of individual synapses may be the underling mechanism of remodeling after the SCI. Understanding the mechanism is important for performing efficient and timely interventions to modulate the pathological and physiological consequences of SCI and to achieve the best recovery outcomes [6,7]. To determine whether SCI leads to bilateral synaptic alterations in the sensory cortex after spinal cord hemisection, we used transcranial two-photon microscopy (thinned skull protocol) and transgenic mice that express YFP predominantly in the layer V pyramidal neurons. We repeatedly imaged individual dendritic branche and spine in the hindlimb sensory cortex after the SCI. We found that remodeling the hindlimb representation of the sensory cortex following spinal cord hemisection occurred bilaterally. This type of remodeling included both eliminating and forming spines simultaneously, and the regions of reorganization in the cortex after the SCI changed over time. The rate of newly formed spines in the contralateral cortex at three days after injury was higher than those of the control group and the ipsilateral cortex in the SCI group. These results demonstrated that soon after the SCI, spine remodeling was performed by increasing spine formation in the contralateral cortex. At two weeks after the SCI, remodeling occurred by increasing both the spine formation and elimination in the contralateral cortex and increasing the spine elimination of the ipsilateral cortex. At the same time, stable spines in bilateral cortices decreased in the SCI group. After that, the remodeling occurred by increasing spine elimination but not spine formation in the bilateral cortices. Synaptic eliminations and formations after the SCI may be critical events for reshaping the dysfunctional cortical connections [29]. From three days to two weeks, the stable rates of bilaterally stable spines in the SCI group decreased. Compared to the control group and the contralateral cortex in the SCI group, the re-emerging rate of eliminated spines in ipsilateral cortex decreased significantly. From two weeks to one month, the stable rates of formed spine in bilateral cortices of the SCI group decreased. These results means that in the early time, spinal cord hemisection made traditional stable spines unstable and led the eliminated spine even more hard to recur especially in the ipsilateral cortex. And it also made the newly formed spines unstable. The low stability of spines may suggest a shrinkage in the structural foundation for information storage, and the plasticity of the dendritic spines offers the cortex the capability to rewire the cortical circuits in response to alternative experiences [45]. The dynamic modulation of the density of spines might indicate that the functional and physiological properties in the sensory cortical circuits are significantly changed by SCI [24]. Such results demonstrate the significant changes that occur in the dendritic spines after SCI and suggest that changes in the inputs to axotomized corticospinal neurons indeed contribute to changes in the cortical hardware of dendritic dendrites [46].

How does spine elimination and formation affect the cortical circuitry? The spine elimination and formation detected here using two-photon fluorescence microscopy reflected synaptic loss and formation [29], which is supported by a pivotal experiment where researchers imaged spine elimination in the sensory cortex *in vivo* using GFP mice and then confirmed it using electron microscopy [33]. According to research on the motor cortex, the state-dependent and state-independent mechanisms may jointly contribute to cortical remodeling immediately after thoracic SCI and this kind of immediate functional remodeling may be one of the mechanisms that lead to long-term cortical reorganization after SCI [6]. Ghosh *et al*. found that several intact dendritic spines may remain functional as axotomized hindlimb corticospinal neurons and can respond to sensory inputs from the forelimb [46]. Corticospinal neurons of the hindlimb motor cortex can sprout new circuits into the cervical spinal cord, accompanied by the appearance of forelimb movements when stimulating hindlimb cortex at three to four weeks after thoracic SCI [45–47].

Why do the differences exist between the bilateral cortices? Different sensory inputs may contribute to different rewiring configurations among the relevant neurons after the SCI. Spinal cord hemisection can affect the two main somatosensory pathways ascending along the spinal cord: the spinothalamic tract and the dorsal columns [47]. The spinothalamic tract, which derives from the paralemniscal pathway, transmits mainly thermal and nociceptive signals to the brain. A left hemisphere hemisection affects the transmission of contralateral thermal and nociceptive sensory to the ipsilateral cortex (the left cortex). Whereas the dorsal columns are derived from the lemniscal pathway, which transmits mainly tactile and proprioceptive signals from the body to the brain. A left hemisphere hemisection affects the transmission of ipsilateral body tactile and proprioceptive sensory information to the contralateral cortex (the right cortex). These two pathways interact with each other at the level of the spinal cord and show some amount of physiological, anatomical, and functional convergence at the thalamic spinal cord level and cortical level in the brain [48]. Different inputs to the bilateral cortices caused by the disruption of the delicate balance between these two pathways in the sensory transmission system which is necessary for transmitting the physiological processing of peripheral signals to the brain, lead to different types of reorganization between the bilateral cortices after spinal cord hemisection.

What role do these different types of reorganization play in the bilateral cortices? Assuming that this is an essential feature of the cortex after injury, these types of reorganization in dendritic spines may be adaptive and beneficial for behavioral recovery, such as in compensatory forelimb use to adapt to thoracic SCI [46,49]. Thus, long-term dendritic spine reorganization may contribute to the recovery of certain motor and sensory functions [46,49–52]. The relevant function decreased significantly after SCI and the eliminated spines may indicate that the nerve is disconnected from the injured and dysfunctional area [29]. The stable and newly formed spines after SCI may allow the injured hindlimb cortical neurons to maintain or form new synapses with other intact functional circuits [53]. On the other hand, this type of cortical reorganization may be maladaptive and excessive or aberrant reorganization of the cortex may induce hallucinatory sensations that do not correspond with objective reality [29]. For example, it may lead to detrimental consequences, such as neuropathic pain [8–11,54], phantom sensation [5,55], and poor cerebral motor or sensory control [1,56–60].

Based on the results reported in this experiment, we suggest that the remodeling processes of synapses in the sensory cortex could be targeted for an intervention to enhance the functional recovery or to manage chronic pain after SCI. Interventions aimed at promoting or decreasing the extent of structural plasticity, prolonging or shortening the time course of reorganization may become important methods for enhancing the functional recovery after SCI. Kim *et al*. demonstrated that the extent of spine remodeling in the motor cortex can be manipulated by interventional methods, such as enriched housing, transplants, and neurotrophin-3 [24]. This highlights the interesting possibility that structural remodeling of dendritic spines in the relevant cortex can be targeted to improve sensory and motor recovery following SCI. For example, by intervening the synaptic remodeling process with the aim of promoting the formation of new synapse or prolonging the time for reorganization, better functional recovery may be achieved. Notwithstanding that the dendritic spine remodels after SCI in the sensory cortex was demonstrated, the specific functional role and the mechanisms of these dendritic spine alterations remain to be explored. There were also some limits in this study and numerous work of more detailed and in-depth studies should be done, for example, it would be more interesting to in vivo explore if somatosensory stimulation (such as temperature, nociception, etc.) or animal exercise could improve the remodeling of synapse in the somatosensory cortex.

## Supporting Information

S1 Table Raw numbers of dendritic spines between bilateral sensory cortices of the hindlimb at three days after spinal cord hemisection. Each digit shows the number of dendritic spines. ‘0d’ means the number of total dendritic spines at the day before the SCI; ‘4d’ means the number of total dendritic spines at three days after the SCI. ‘Stable’ means the number of stable dendritic spines. ‘Elinination’ means the number of eliminated dendritic spines. ‘Formation’ means the number of new formed dendritic spines. Here showes the raw number of dendritic spines from bilateral cortices in the control group.
(DOC)

S2 Table Raw numbers of dendritic spines between bilateral sensory cortices of the hindlimb at two weeks after spinal cord hemisection. Each digit shows the number of dendritic spines. ‘0d’ means the number of total dendritic spines at the day before the SCI. ‘2w’ means the number of total dendritic spines at two weeks after the SCI. ‘Stable’ means the number of stable dendritic spines. ‘Elinination’ means the number of eliminated dendritic spines. ‘Formation’ means the number of new formed dendritic spines. One mouse’s ipsilateral skull of the SCI group was damaged in the third skull-thinning process.
(DOC)

S3 Table Raw numbers of dendritic spines between bilateral sensory cortices of the hindlimb at one month after spinal cord hemisection. Each digit shows the number of dendritic spines. ‘0d’ means the number of total dendritic spines at the day before the SCI. ‘1m’ means the number of total dendritic spines at one month after the SCI. ‘Stable’ means the number of stable dendritic spines. ‘Elinination’ means the number of eliminated dendritic spines. ‘Formation’ means the number of new formed dendritic spines.
(DOC)
