# Supplementary material for: Remodeling the Dendritic Spines in the Hindlimb Representation of the Sensory Cortex after Spinal Cord Hemisection in Mice
Source: PLoS One. 2015 Jul 1;10(7):e0132077. doi: 10.1371/journal.pone.0132077 (PMC4489092; doi:10.1371/journal.pone.0132077)
Supplement: S2 Table — Each digit shows the number of dendritic spines. ‘0d’ means the number of total dendritic spines at the day before the SCI. ‘2w’ means the number of total dendritic spines at two weeks after the SCI. ‘Stable’ means the number of stable dendritic spines. ‘Elinination’ means the number of eliminated dendritic spines. ‘Formation’ means the number of new formed dendritic spines. One mouse’s ipsilateral skull of the SCI group was damaged in the third skull-thinning process. (DOC) [file pone.0132077.s002.doc]

**S2 Table. Raw numbers of dendritic spines between bilateral sensory cortices of the hindlimb at two weeks after spinal cord hemisection**

| **Control** | | | | | **Ipsilateral** | | | | | **Contralateral** | | | | |
| --- | --- | --- | --- | --- | --- | --- | --- | --- | --- | --- | --- | --- | --- | --- |
| **0d** | **2w** | **Stable** | **Elimination** | **Formation** | **0d** | **2w** | **Stable** | **Elimination** | **Formation** | **0d** | **2w** | **Stable** | **Elimination** | **Formation** |
| 155 | 155 | 143 | 12 | 12 | 159 | 143 | 127 | 32 | 16 | 156 | 134 | 125 | 31 | 9 |
| 156 | 150 | 134 | 22 | 16 | 184 | 178 | 168 | 16 | 9 | 175 | 163 | 139 | 36 | 23 |
| 188 | 157 | 142 | 46 | 16 | 150 | 134 | 125 | 25 | 10 | 175 | 169 | 145 | 30 | 23 |
| 176 | 176 | 158 | 18 | 18 | 167 | 151 | 139 | 28 | 13 | 167 | 150 | 132 | 35 | 18 |
| 189 | 187 | 172 | 17 | 15 | 188 | 181 | 161 | 27 | 20 | 165 | 143 | 131 | 34 | 13 |
| 160 | 159 | 146 | 14 | 13 | 162 | 142 | 130 | 32 | 12 | 166 | 157 | 143 | 23 | 14 |
| 178 | 158 | 155 | 23 | 2 | 165 | 158 | 140 | 25 | 18 | 197 | 194 | 171 | 26 | 22 |
| 190 | 177 | 165 | 25 | 12 | 163 | 148 | 136 | 27 | 12 | 154 | 148 | 133 | 21 | 15 |
| 185 | 176 | 166 | 19 | 9 | - | - | - | - | - | 166 | 163 | 145 | 21 | 18 |
| 180 | 169 | 157 | 23 | 12 | - | - | - | - | - | - | - | - | - | - |
| 178 | 150 | 144 | 34 | 6 | - | - | - | - | - | - | - | - | - | - |
| 173 | 159 | 141 | 32 | 18 | - | - | - | - | - | - | - | - | - | - |
| 178 | 178 | 154 | 24 | 23 | - | - | - | - | - | - | - | - | - | - |
| 167 | 175 | 157 | 10 | 18 | - | - | - | - | - | - | - | - | - | - |
| 169 | 158 | 143 | 26 | 16 | - | - | - | - | - | - | - | - | - | - |
| 162 | 147 | 142 | 20 | 5 | - | - | - | - | - | - | - | - | - | - |
| 178 | 167 | 158 | 20 | 10 | - | - | - | - | - | - | - | - | - | - |
| 165 | 154 | 145 | 20 | 9 | - | - | - | - | - | - | - | - | - | - |

Each digit shows the number of dendritic spines. ‘0d’ means the number of total dendritic spines at the day before the SCI. ‘2w’ means the number of total dendritic spines at two weeks after the SCI. ‘Stable’ means the number of stable dendritic spines. ‘Elinination’ means the number of eliminated dendritic spines. ‘Formation’ means the number of new formed dendritic spines. One mouse’s ipsilateral skull of the SCI group was damaged in the third skull-thinning process.
