# Supplementary material for: Remodeling the Dendritic Spines in the Hindlimb Representation of the Sensory Cortex after Spinal Cord Hemisection in Mice
Source: PLoS One. 2015 Jul 1;10(7):e0132077. doi: 10.1371/journal.pone.0132077 (PMC4489092; doi:10.1371/journal.pone.0132077)
Supplement: S3 Table — Each digit shows the number of dendritic spines. ‘0d’ means the number of total dendritic spines at the day before the SCI. ‘1m’ means the number of total dendritic spines at one month after the SCI. ‘Stable’ means the number of stable dendritic spines. ‘Elinination’ means the number of eliminated dendritic spines. ‘Formation’ means the number of new formed dendritic spines. (DOC) [file pone.0132077.s003.doc]

**S3 Table. Raw numbers of dendritic spines between bilateral sensory cortices of the hindlimb at one month after spinal cord hemisection**

| **Control** | | | | | **Ipsilateral** | | | | | **Contralateral** | | | | |
| --- | --- | --- | --- | --- | --- | --- | --- | --- | --- | --- | --- | --- | --- | --- |
| **0d** | **1m** | **Stable** | **Elimination** | **Formation** | **0d** | **1m** | **Stable** | **Elimination** | **Formation** | **0d** | **1m** | **Stable** | **Elimination** | **Formation** |
| 155 | 172 | 144 | 11 | 28 | 167 | 155 | 134 | 33 | 21 | 175 | 170 | 145 | 30 | 25 |
| 185 | 171 | 155 | 30 | 16 | 163 | 141 | 134 | 29 | 7 | 149 | 128 | 106 | 43 | 22 |
| 149 | 144 | 129 | 20 | 15 | 198 | 171 | 152 | 46 | 19 | 169 | 144 | 131 | 38 | 13 |
| 160 | 163 | 141 | 19 | 22 | 168 | 162 | 143 | 25 | 19 | 197 | 182 | 157 | 40 | 24 |
| 186 | 163 | 156 | 30 | 8 | 188 | 170 | 148 | 40 | 22 | 166 | 166 | 146 | 20 | 21 |
| 191 | 168 | 154 | 37 | 15 | 150 | 137 | 121 | 29 | 16 | 167 | 137 | 121 | 46 | 16 |
| 178 | 166 | 141 | 37 | 25 | 155 | 123 | 117 | 38 | 7 | 165 | 142 | 127 | 38 | 15 |
| 182 | 178 | 162 | 20 | 16 | 159 | 145 | 126 | 33 | 19 | 154 | 141 | 120 | 34 | 21 |
| 173 | 181 | 157 | 16 | 24 | - | - | - | - | - | - | - | - | - | - |
| 184 | 183 | 160 | 24 | 23 | - | - | - | - | - | - | - | - | - | - |
| 169 | 155 | 132 | 37 | 23 | - | - | - | - | - | - | - | - | - | - |
| 176 | 174 | 155 | 21 | 19 | - | - | - | - | - | - | - | - | - | - |
| 193 | 164 | 154 | 39 | 10 | - | - | - | - | - | - | - | - | - | - |
| 166 | 136 | 129 | 37 | 8 | - | - | - | - | - | - | - | - | - | - |
| 154 | 147 | 128 | 26 | 19 | - | - | - | - | - | - | - | - | - | - |
| 172 | 156 | 145 | 27 | 11 | - | - | - | - | - | - | - | - | - | - |

Each digit shows the number of dendritic spines. ‘0d’ means the number of total dendritic spines at the day before the SCI. ‘1m’ means the number of total dendritic spines at one month after the SCI. ‘Stable’ means the number of stable dendritic spines. ‘Elinination’ means the number of eliminated dendritic spines. ‘Formation’ means the number of new formed dendritic spines.
